# Efficacy of regular prophylaxis with a plasma-derived von Willebrand factor/factor VIII concentrate with a 1:1 activity ratio in reducing heavy menstrual bleeding in girls/women with von Willebrand disease

**DOI:** 10.1016/j.xagr.2025.100501

**Published:** 2025-05-02

**Authors:** Csongor Kiss, Zoltan Boda, Claudia Djambas Khayat, Ana Boban, Leonid Dubey, Robert F. Sidonio Jr

**Affiliations:** 1 Division of Pediatric Hematology-Oncology, Department of Pediatrics, Faculty of Medicine, University of Debrecen, Debrecen, Hungary (Kiss); 2 Division of Hematology, Department of Internal Medicine, Faculty of Medicine, University of Debrecen, Debrecen, Hungary (Boda); 3 Hotel Dieu de France Hospital, Saint Joseph University, Beirut, Lebanon (Khayat); 4 Department of Internal Medicine, University Hospital Centre Zagreb, Zagreb University School of Medicine, Zagreb, Croatia (Boban); 5 Communal Nonprofit Enterprise “Western Ukrainian Specialized Children’s Medical Center” of Lviv Regional Council, Lviv, Ukraine (Dubey); 6 Department of Pediatrics, Emory University School of Medicine, Atlanta, GA, (Sidonio)

**Keywords:** clinical trial, women’s health, bleeding and clotting, inherited bleeding disorder, replacement therapy

## Abstract

**Background:**

Heavy menstrual bleeding is the dominant symptom in girls/women with von Willebrand disease (VWD), affecting ∼80 to 90%. Female patients with VWD are typically diagnosed later than males, despite the disproportionate impact of heavy menstrual bleeding. Although heavy menstrual bleeding has a substantial impact on patients, it is commonly undertreated in part because of the potential multifactorial causes in young girls/women, imprecise definition, and exclusion of this bleeding type as an indication for prophylaxis in previous studies. The WIL-31 study showed that prophylaxis with wilate (a plasma-derived von Willebrand factor/factor VIII concentrate with a 1:1 activity ratio) is highly efficacious in reducing bleeding rates in adults and children with VWD of all types. The impact of wilate prophylaxis on heavy menstrual bleeding was evaluated as an exploratory endpoint since it was not included as part of the primary endpoint.

**Objective(s):**

To investigate the efficacy of regular prophylaxis with wilate in reducing the incidence of heavy menstrual bleeding in girls/women with VWD who had previously been treated on-demand.

**Study design:**

WIL-31 (NCT04052698) was a prospective, non-controlled, international, multicenter Phase 3 trial that enrolled male/female patients, aged ≥ 6 years old with VWD. Prior to entering the WIL-31 study, all patients received on-demand treatment with a von Willebrand factor-containing product during a 6-month, prospective, observational, run-in study (WIL-29). Patients in WIL-31 received wilate prophylaxis 2–3 times per week at a dose of 20–40 IU/kg for 12 months. Prophylaxis was not tailored to time with menstruation. Heavy menstrual bleeding was defined as any menstrual bleed that impeded the ability to perform daily activities during menstrual periods. Criteria for heavy menstrual bleeding included changing pads more frequently than hourly, menstrual bleeding lasting 7 or more days, or the presence of clots > 1 cm, combined with a history of flooding or a Pictorial Blood Assessment Chart score ≥ 185.

**Results:**

Of the 33 patients evaluated in the study, 14 (42%) were female, of whom 5 (15%) were of childbearing age (13–43 years old). One of these had VWD type 1, the other 4 had VWD type 3. Prophylaxis with wilate reduced the mean annualized heavy menstrual bleeding rate by 75% compared with on-demand treatment (2.4 vs 9.7 in WIL-31 and WIL-29, respectively). During 6 months of on-demand treatment, all 5 female patients experienced at least 1 heavy menstrual bleed, whereas during 12 months of prophylaxis, 3 (60%) experienced no heavy menstrual bleeding episodes. During on-demand treatment, 5 patients experienced 26 heavy menstrual bleeding episodes, 3 (12%) of which required additional treatment. Under prophylaxis, 2 patients experienced 12 heavy menstrual bleeding episodes, of which only 1 (8%) impeded daily activity and none required additional treatment.

**Conclusions:**

In these exploratory analyses, wilate prophylaxis given 2–3 times a week without menstruation coordination was efficacious in reducing heavy menstrual bleeding in girls/women with VWD compared with on-demand treatment. Long-term prophylaxis has the potential to play a major role in improving the care and reducing the disease burden for girls/women with VWD.

## Graphical abstract

**Figure:**
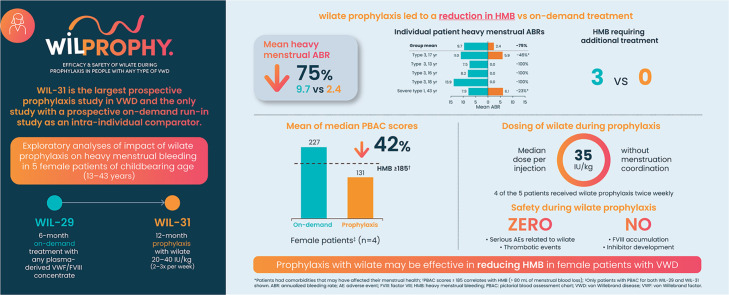

**AJOG Global Reports at a Glance**

**Why was this study conducted?**

Heavy menstrual bleeding is the most common symptom affecting girls/women with von Willebrand disease (VWD) with substantial impacts on quality of life and long-term complications, yet there is a lack of published data in this population.

**Key findings**

The Phase 3 WIL-31 study is the largest prospective study specifically investigating the efficacy and safety of von Willebrand factor/factor VIII prophylaxis with 1:1 ratio in patients with VWD. Heavy menstrual bleeding reduced by 75% during prophylaxis compared with prior on-demand treatment despite comorbidities.

**What does this add to what is known?**

Heavy menstrual bleeding is measured separately from other bleeding events and thus poorly described. Prophylaxis with wilate may be effective in reducing menstrual blood loss in girls/women with VWD.

## Introduction

Heavy menstrual bleeding (HMB) is defined as excessive menstrual blood loss that interferes with a female’s (defined here as including transgender people or those with the ability to menstruate) physical, emotional, social, and material quality of life (QoL).^1^ HMB is under-recognized, under-treated, and may affect up to 50% of females of reproductive age by population-based estimates, whereas data from healthcare systems indicate a prevalence of 3–5%.^2^ Low diagnosis rates have been attributed to stigmas surrounding HMB among patients and healthcare professionals (HCPs) coupled with a lack of awareness from patients surrounding what constitutes abnormal bleeding.^3^ The predominance of male patients with hemophilia may lead to the perception among non-specialists that bleeding disorders rarely affect girls/women, causing a diagnosis bias.^4^

HMB has many potential causes, broadly divided into structural causes, such as polyps and adenomyosis, and non-structural causes such as ovulatory and coagulation disorders.^5^^,^^6^ Estimates from retrospective studies indicate that 10–62% of adolescents with HMB may have an underlying inherited bleeding disorder, such as von Willebrand disease (VWD).^7^ VWD is the most common inherited bleeding disorder, affecting approximately 0.6–1.3% of the population.^8^ It stems from quantitative and qualitative defects in von Willebrand factor (VWF), leading to increased bleeding symptoms.^9^ Although VWD affects males and females with equal frequency, females are disproportionately impacted. HMB is the most commonly reported symptom in girls/women, estimated across multiple studies to affect 61–100% of girls/women of reproductive age with VWD.^10^ It is recommended to screen for VWD in adolescents presenting with HMB, adults with HMB without any gynecologic cause, and adults with HMB undergoing hysterectomy. However, many patients are not screened.^11^ It is a topic of active uncertainty whether to screen patients presenting with HMB close to menarche with no family history of a bleeding disorder.^12^

Current guidelines recommend consideration of hormonal therapy in girls/women with VWD who do not wish to conceive and to consider tranexamic acid in those who wish to conceive.^13^ Patients not responding to these treatments or with contraindications should be treated with von Willebrand factor/factor VIII (VWF/FVIII).^14^ In some patients, VWF/FVIII prophylaxis may be an alternative to surgical control to mitigate HMB symptoms.^15^ For many patients, prophylaxis may be preferable to on-demand treatment to more effectively manage HMB, prevent complications, prevent unnecessary procedures, and reduce the frequency of other bleed types. Despite this, there are limited data on the efficacy of prophylaxis for HMB in girls/women with VWD. wilate is a plasma-derived factor concentrate containing VWF and factor VIII (pdVWF/FVIII) in a physiological 1:1 activity ratio that is indicated in VWD patients older than 6-years-old for the prevention and treatment of bleeds and perioperative management of bleeding.^16^^,^^17^ The Phase 3 WIL-31 study (NCT04052698) showed that wilate prophylaxis was highly efficacious in reducing bleeding rates in adults and children with VWD of all types. The primary endpoint was met, with an 84% reduction in annualized bleeding rate (ABR).^18^ The impact of wilate prophylaxis on HMB was evaluated in the study as an exploratory endpoint of the WIL-31 study. We report here the efficacy of regular wilate prophylaxis vs prior on-demand treatment to manage HMB in girls/women with VWD.

## Material and methods

WIL-31 (NCT04052698; WILPROPHY) was a prospective, non-controlled, international, multicenter Phase 3 trial that enrolled male/female patients aged ≥ 6 years with VWD type 1 (VWF:RCo < 30 IU/dL), type 2 (except 2N) or type 3. Prior to entering the WIL-31 study, all patients had received on-demand treatment with any pdVWF/FVIII concentrate during a 6-month, prospective, observational, run-in study (WIL-29); patients who experienced at least 6 bleeding events (BEs) during WIL-29, excluding menstrual bleeds, with at least 2 of these BEs treated with a VWF-containing product, were eligible to enter WIL-31. Patients in WIL-31 received wilate prophylaxis 2–3 times per week at a dose of 20–40 IU/kg for 12 months and dosing was not tailored to the timing of menstruation. For further details, please refer to the primary publication.^18^ Female patients agreed to use adequate birth control measures and were made aware that hormonal contraception use should remain unchanged throughout the study. Trans men assigned female at birth would have been eligible for inclusion in these analyses, but none were enrolled. The study was performed in accordance with the Declaration of Helsinki and the respective local regulations. Voluntarily given, fully informed written and signed consent was obtained from patients (or their legal guardians) before any study-related procedures were conducted.

VWF and FVIII activity levels were measured by the VWF:RCo, chromogenic and 1-stage assays, respectively, at baseline and after 1, 2, 3, 6, 9 and 12 months of treatment. Blood samples were taken within 60 min before and 60 ± 5 min after injection.

Exploratory analyses of WIL-31 included comparison of the heavy menstrual ABR (HMABR) in WIL-31 vs WIL-29. HMB was defined as any menstrual bleed that impeded the ability to perform daily activities (such as work, housework, exercise, or social activities) during menstrual periods. Criteria for HMB included changing pads more frequently than hourly, menstrual bleeding lasting 7 or more days, or the presence of clots > 1 cm combined with a history of flooding or a Pictorial Blood Assessment Chart (PBAC) score ≥ 185. PBAC scores were reviewed from patient diaries at each study visit.

### Analyses

Analyses were performed on data from a subgroup of female participants of childbearing age with confirmed VWD who completed WIL-31 and received 12 months of prophylaxis. Mean and individual HMABRs and changes in PBAC score were assessed. All statistical analyses were descriptive.

## Results

### Patient disposition and demographics

Of the 33 patients enrolled in the WIL-31 study, 42% (14/33) were female, and 7 were of childbearing age (aged 13–43 years). Two of these female patients discontinued wilate early, 1 due to mild chest tightness on 3 occasions (discontinued after 2 exposure days) and the other due to moderate hypersensitivity (discontinued after 6 exposure days). The results presented here are therefore from the subgroup of 5 female patients of childbearing age who received 12 months of wilate prophylaxis. One female patient had VWD type 1 and 4 had VWD type 3. The demographics and baseline characteristics of these patients are summarized in Table 1**.**

**Table 1:** Demographics and baseline characteristics of female patients of childbearing age during WIL-31

Characteristic	Patients (N = 5)
**VWD type, n (%)**	
Severe Type 1	1 (20%)
Type 2A	0 (0%)
Type 3	4 (80%)
**Age at screening, years:** median (range)	17 (13–43)
**Weight at screening, kg:** median (range)	64.5 (53–94)
**Height at screening, cm:** median (range)	167 (163–170)
**Race, n (%)**	
Caucasian	5 (100%)
**Blood type, n (%)**	
A	2 (40%)
B	0 (0%)
AB	0 (0%)
O	3 (60%)
**Family history of VWD, n (%)**	2 (40%)

Two patients had comorbidities that may have affected their menstrual health. One patient was affected by ongoing polycystic ovaries, and 1 by ongoing hypothyroidism, obesity, anemia, type 2 diabetes, and hypertension. All patients started hormonal birth control before WIL-29 began and continued taking their medication for the duration of both studies **(**Table 2**)**. Four patients were taking combined estrogen/progesterone oral contraceptives, the remaining patient was taking a progesterone-only oral contraceptive.

**Table 2:** Contraceptive use in female patients of childbearing age during WIL-31

Patient characteristics	Contraceptive	Frequency
Severe VWD type 1, 43 yr	Ethinylestradiol; levonorgestrel	Daily
VWD type 3, 18 yr	Dienogest; ethinylestradiol	Daily
VWD type 3, 16 yr	Drospirenone; ethinylestradiol betadex clathrate	Daily
VWD type 3, 13 yr	Norethisterone acetate	Daily
VWD type 3, 17 yr	Drospirenone; ethinylestradiol	Daily

### Heavy menstrual bleeding

**Figure 1:**
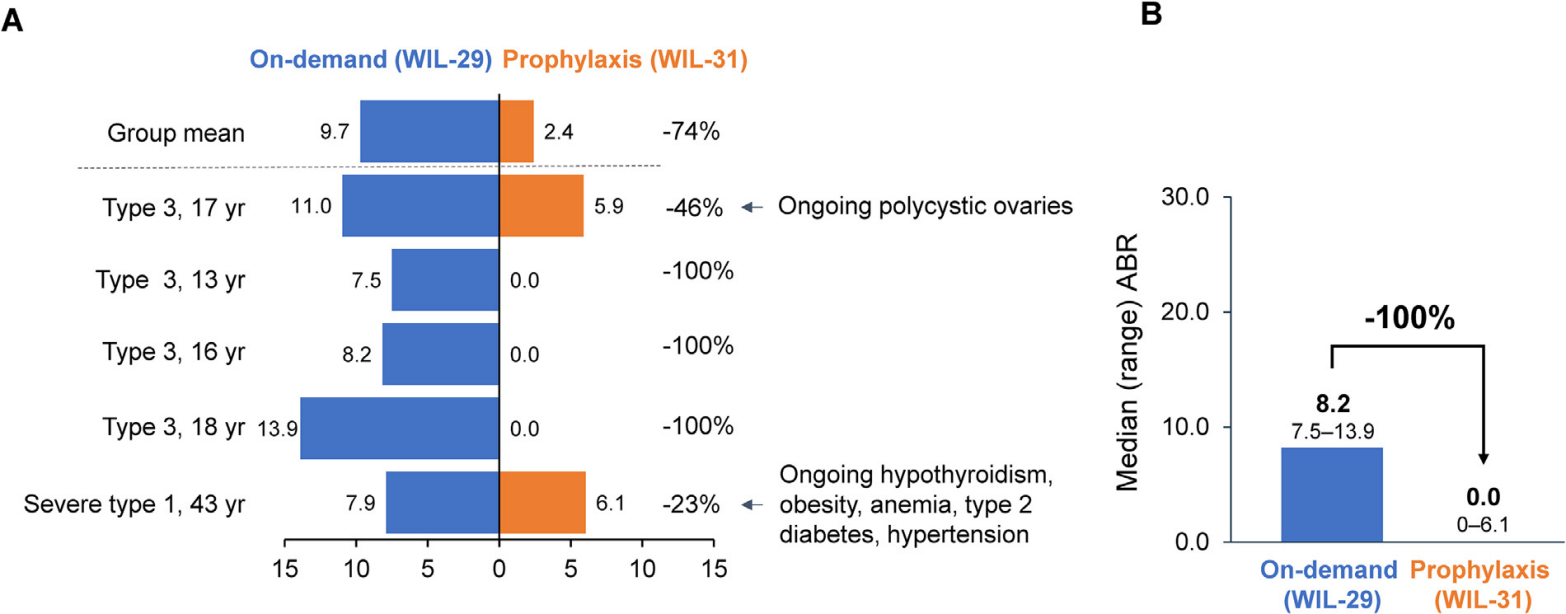
Individual (A) and median (B) patient heavy menstrual ABR for female patients of childbearing age during WIL-29 and WIL-31 (N = 5) ABR: Annualized bleeding rate; yr: years of age.

During 6 months of on-demand treatment, all 5 patients experienced at least 1 HMB episode, with a collective total of 26 HMB episodes. During 12 months of prophylaxis, 2 patients (40%) experienced 12 HMB episodes; both patients were adults (aged ≥ 17 years) and had comorbidities that may have affected their menstrual health. Prophylaxis with wilate reduced the mean HMABR by 75% compared with on-demand treatment (2.4 vs 9.7 during WIL-31 and WIL-29, respectively) **(**Figure 1A). Prophylaxis reduced the median HMABR by 100% compared with on-demand treatment (0.0 vs 8.2 during WIL-31 and WIL-29 respectively) **(**Figure 1B).

Kiss. Prophylaxis with pdVWF/FVIII Reduces Heavy Menstrual Bleeding in VWD. Am J Obstet Gynecol Glob Rep 2025.

**Figure 2:**
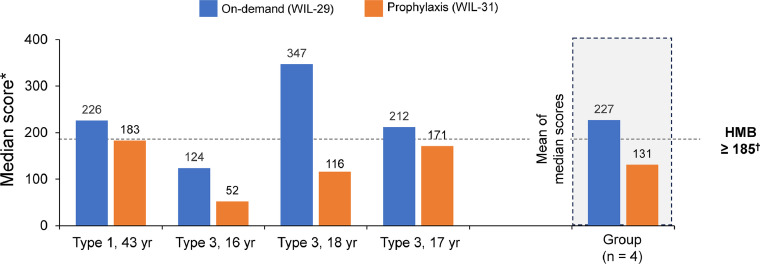
Individual median and group mean PBAC scores for female patients of childbearing age during WIL-29 and WIL-31 (n = 4) HMB: Heavy menstrual bleeding; PBAC: Pictorial blood assessment chart; yr: Years. *Only patients with PBAC for both WIL-29 and WIL-31 shown. ^†^PBAC scores ≥ 185 correlates with HMB (> 80 mL of menstrual blood loss).

During the on-demand period, 3 (12%) HMB episodes required further treatment, 1 with Haemate (2 injections of 26 IU/kg) and concomitant desmopressin, 1 with desmopressin, and 1 with tranexamic acid. During prophylaxis, no HMB episode required additional treatment, and only 1 episode impeded daily activity in these patients. The mean of median intra-individual PBAC scores was reduced by 42% during prophylaxis compared with on-demand treatment for 4 patients with available data (131 vs 227, in WIL-31 and WIL-29 respectively) **(**Figure 2**).**

Kiss. Prophylaxis with pdVWF/FVIII Reduces Heavy Menstrual Bleeding in VWD. Am J Obstet Gynecol Glob Rep 2025.

### Dosing and prophylactic regimen

The median (range) wilate dose for prophylaxis was 34.5 (24.3–39.2) IU/kg per injection. The median (range) weekly prophylactic dose was 67.8 (48.5–94.9) IU/kg. The weekly doses of the 2 patients who had HMB episodes during prophylaxis were 48.5 IU/kg and 70.1 IU/kg.

Four patients received wilate prophylaxis twice weekly. The remaining patient received twice weekly wilate prophylaxis for 6 months, which was increased to 3 times weekly due to left- and right-ankle arthropathy. This patient experienced 10 traumatic bleeds (5 nose; 3 right ankle, 1 right knee, 1 cutaneous left index finger) and 1 spontaneous bleed (right ankle joint) while receiving prophylaxis twice weekly and 2 traumatic bleeds (right ankle joint) while receiving prophylaxis 3 times weekly.

### Safety

**Figure 3:**
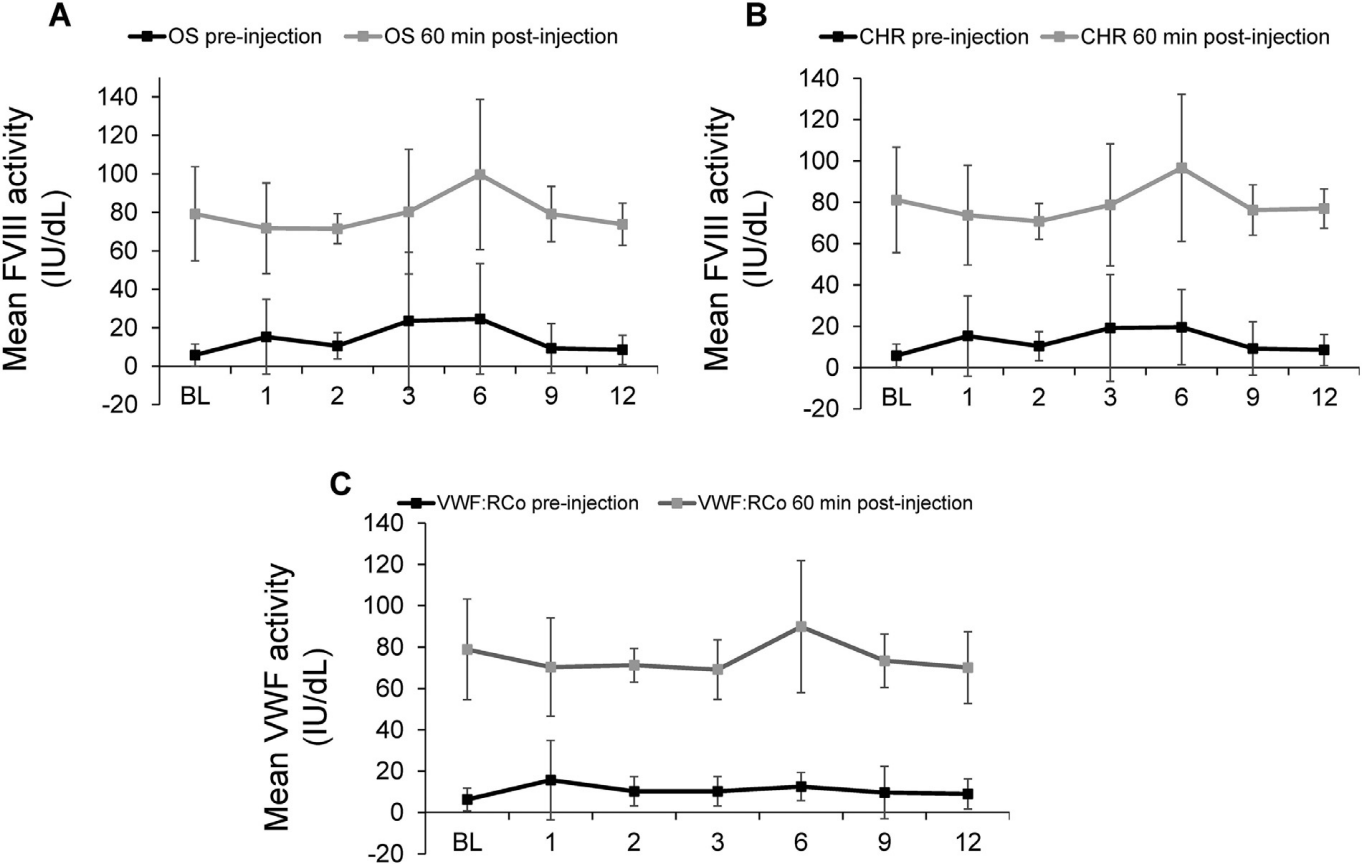
Mean FVIII one-stage (A), chromogenic (B) and VWF:RCo (C) activity for female patients of childbearing age during WIL-31 CHR: chromogenic; OS: one-stage; VWF:RCo: Von Willebrand Ristocetin Cofactor

VWF and FVIII activity levels were stable throughout the study, showing no accumulation **(**Figure 3**)**. The mean (SD) VWF activity was 6.2 (2.7) IU/dL at baseline and 5.8 (1.8) IU/dL at 12 months pre-injection and 66.2 (22.5) IU/dL and 42.6 (15.8) IU/dL post-injection. The values for FVIII measured by chromogenic assay were 5.8 (5.6) IU/dL at baseline and 8.5 (7.5) IU/dL at 12 months pre-injection and 81.2 (25.5) at baseline and 77.0 (9.5) IU/dL at 12 months post-injection. The corresponding values for FVIII activity measured by the 1-stage assay were 6.4 (7.4) IU/dL and 11.7 (11.5) IU/dL pre-injection and 81.4 (29.7) IU/dL and 79.1 (16.4) IU/dL post-injection respectively. No thrombotic events were observed, and no patient developed an inhibitor to VWF or FVIII during the study.

Kiss. Prophylaxis with pdVWF/FVIII Reduces Heavy Menstrual Bleeding in VWD. Am J Obstet Gynecol Glob Rep 2025.

## Comments

### Principal findings

The Phase 3 WIL-31 study is the largest prospective study specifically investigating the efficacy and safety of VWF prophylaxis in patients with VWD. Prophylaxis with wilate was efficacious and well tolerated in adults and children with all types of VWD.^18^ Here we report that wilate prophylaxis, irrespective to the timing of menstruation, reduced HMABR by 75% compared with prior on-demand treatment in girls/women of childbearing age. The severity of menstrual bleeding measured by PBAC scores improved by 42%. The median wilate dose for prophylaxis was 34.5 (24.3–39.2) IU/kg per injection, with 4 of the 5 patients receiving wilate prophylaxis twice weekly.

### Results in the context of what is known

Hormonal and non-hormonal treatments for HMB are ineffective or cannot be tolerated by up to 30% of girls/women.^19^ Despite the impact of HMB on the health and wellbeing of girls/women with VWD, published data on the efficacy of prophylaxis for HMB are limited. Many studies have included girls/women of childbearing age but did not measure or report HMB outcomes.^20^, ^21^, ^22^, ^23^, ^24^

The scarce data that are available support the use of VWF prophylaxis to reduce HMB in girls/women with VWD, however, with less focus on the timing of administration of concentrate. Results from the VWD Prophylaxis Network indicate that VWF prophylaxis is effective for reducing HMB. Compared with prior on-demand treatment, prophylaxis significantly reduced HMABR for 9 female participants with all types of VWD (median 9.6 vs 0.0, *p* = 0.008)^25^ and in 4 female participants reduced the number of menstrual cycles with reported HMB from 12 to 4 per year.^26^ In a cohort study of 32 patients who received 12-month pdVWF/FVII prophylaxis, prophylaxis prevented recurrent BEs in 7 female participants who initiated prophylaxis due to HMB.^27^ During the SWIFT-VWD trial, 1 female participant received once-monthly prophylaxis with pdVWF/FVIII for HMB. This participant experienced 18 BEs during 12 months of on-demand treatment vs 1 BE during 12 months of prophylaxis, but the details of bleed type were not provided.^28^ An unplanned interim analysis of the VWDmin study comparing prophylaxis with recombinant VWF (rVWF) to tranexamic acid for HMB found that rVWF was non-superior in patients with mild or moderate VWD.^19^ Neither rVWF nor tranexamic acid met the primary endpoint of reducing PBAC scores to normal levels, although both treatments significantly reduced PBAC scores.

### Clinical implications

HMB imposes a substantial disease burden on girls/women with bleeding disorders, and commonly prescribed treatments are inadequate for many. Although hormonal contraceptives are recommended to reduce HMB, only 1 hormonal contraceptive (containing estradiol valerate and dienogest) has been approved by the US FDA to treat HMB.^29^ Among girls/women with VWD, HMB is the most common cause of outpatient visits (22.5 vs 5.8 per 1000 person years for VWD vs control) and is a common cause of hospitalization (3.5/1000 person years).^30^ Girls/women with VWD are more likely to undergo gynecologic surgery, including hysterectomy, than age-matched controls^31^; 11% of girls/women with bleeding disorders attending a Hemophilia Treatment Center (HTC) had undergone hysterectomy specifically to reduce HMB.^15^ Further, girls/women with VWD are more likely to have intraoperative and postoperative bleeding following hysterectomy.^32^ Adolescents with HMB-related bleeding disorders have a higher prevalence of depression and anxiety than adolescents with HMB and no coagulation disorder.^11^ Despite this, girls/women with VWD and HMB remain undertreated; of 1321 female patients with VWD seen at HTCs from 2011 to 2014, 62% (816/1321) had HMB but VWF was given during menstruation to treat just 13 patients (1.6%).^33^ 73% (138/190) of adults faced a 10-year delay in obtaining a VWD diagnosis after the onset of HMB.^34^ A national database study found that only 8% of adolescents with HMB and 16% of adolescents with severe HMB were screened for VWD.^35^ Factors such as pregnancy, stress, and estrogen^11^ can alter VWF levels, necessitating repeat testing for girls/women suspected of having VWD.

### Research implications

Further research is needed to ensure equitable access to care for girls/women with VWD. Faster diagnosis of VWD and referral for treatment has been identified by an expert panel as fundamental in addressing the inequity in provision of care for girls/women in the UK and Ireland.^4^ VWF levels vary due to external factors such as stress and pregnancy, potentially delaying diagnosis by laboratory tests. As such, genetic diagnosis has the potential to improve diagnostic rates by allowing for accurate testing of potential patients, as well as accurate and rapid identification of potentially affected family members.^29^ For girls/women with VWD who require treatment with VWF concentrates, further research is needed to develop the optimum treatment plan. Guidelines do not specify the dose or regimen which should be used for prophylaxis to reduce HMB. Studies of other VWF concentrates have included girls/women who were treated only during menstruation.^28^ This analysis provides further evidence for long-term continuous prophylaxis for the prevention of HMB. Future studies, such as the ongoing EMPOWER trial of wilate prophylaxis for HMB in girls/women with VWD (NCT06205095, https://www.clinicaltrials.gov/study/NCT06205095), will further strengthen the existing knowledge base.

### Strengths and limitations

The Phase 3 WIL-31 study was the largest prospective study with an on-demand run in study as an intra-individual comparator investigating the efficacy and safety of VWF prophylaxis in patients with VWD and provides evidence for the use of wilate prophylaxis to reduce HMB in girls/women with VWD. Despite being the largest such study, the number of female participants of child-bearing age was rather small, and none of the comparisons were statistically powered. As WIL-31 was not specifically designed to examine HMB, additional relevant information for HMB including underlying causes and comorbidities were not documented in detail. In addition, some data points were not collected during WIL-31 that may have provided useful insights, such as ferritin levels, which are the preferred measurement to monitor iron-deficiency, a common consequence of HMB.^36^ Future prospective studies with a larger sample size will be needed to confirm our findings.

## Conclusions

In these exploratory analyses, prophylaxis with pdVWF/FVIII with 1:1 ratio was efficacious in reducing HMB compared with on-demand treatment in girls/women with VWD. None of the cases of HMB during prophylaxis was severe enough to require additional treatment. Long-term prophylaxis has the potential to play a major role in improving the care and reducing the disease burden for girls/women with VWD.

## CRediT authorship contribution statement

**Csongor Kiss:** Writing – review & editing, Writing – original draft, Conceptualization. **Zoltan Boda:** Writing – review & editing, Writing – original draft, Conceptualization. **Claudia Djambas Khayat:** Writing – review & editing, Writing – original draft, Conceptualization. **Ana Boban:** Writing – review & editing, Writing – original draft, Conceptualization. **Leonid Dubey:** Writing – review & editing, Writing – original draft, Conceptualization. **Robert F. Sidonio Jr:** Writing – review & editing, Writing – original draft, Conceptualization.
